# Multi‐Functional Bio‐HJzyme: Revolutionizing Diabetic Skin Regeneration with its Glucose‐Unlocked Sterilization and Programmed Anti‐Inflammatory Effects

**DOI:** 10.1002/advs.202300986

**Published:** 2023-05-10

**Authors:** Miaomiao He, Zuyao Wang, Hang Yang, Qiancun Wang, Danni Xiang, Xinyan Pang, Yau Kei Chan, Dan Sun, Guangfu Yin, Weizhong Yang, Yi Deng

**Affiliations:** ^1^ College of Biomedical Engineering School of Chemical Engineering Sichuan University Chengdu 610065 China; ^2^ Department of Ophthalmology The University of Hong Kong Hong Kong SAR 999077 China; ^3^ State Key Laboratory of Polymer Materials Engineering Sichuan University Chengdu 610065 China; ^4^ Department of Mechanical Engineering The University of Hong Kong Hong Kong SAR 999077 China; ^5^ Advanced Composite Research Group (ACRG) School of Mechanical and Aerospace Engineering Queen's University Belfast Belfast BT9 5AH UK

**Keywords:** antibacterial, anti‐inflammation, bio‐heterojunction, cutaneous regeneration, glucose‐unlocked

## Abstract

Antibacterial dynamic therapy (ADT) triggered by reactive oxygen species (ROS) is promising for diabetic infectious disease treatment. However, the limited local O_2_/H_2_O_2_ production and post‐treatment inflammation remain long‐standing issues. To address these challenges, a novel H_2_‐evolving bio‐heterojunction enzyme (Bio‐HJzyme) consisting of graphite‐phase carbon nitride/copper sulfide (CN/Cu_2−*x*_S) heterojunction and glucose oxidase (GO*x*) is created. The Bio‐HJzyme offers glutathione peroxidase (GP*x*), peroxidase (POD), and catalase (CAT) mimetic activities; provides anti‐pathogen properties via programmed light activation; and effectively promotes diabetic wound healing. Specifically, its GP*x*‐mimetic activity and the presence of GO*x* significantly enhance the yield of H_2_O_2_, which can be catalyzed through POD‐mimetic activity to produce highly germicidal •OH. The H_2_O_2_ can also be catalyzed to H_2_O and O_2_, assisted by the CAT‐mimetic activity. The catalyzed products can then be catalyzed into germicidal •OH and •O_2_^−^ under NIR light irradiation, giving enhanced ADT. Further, CN can split water to form H_2_ under solar light, which dramatically suppresses the inflammation caused by excessive ROS. In vivo evaluation confirms that Bio‐HJzyme promotes the regeneration of diabetic infectious skin through killing bacteria, enhancing angiogenesis, promoting wound bed epithelialization, and reinforcing anti‐inflammatory responses; hence, providing a revolutionary approach for diabetic wounds healing.

## Graphical abstract

A Bio‐HJzyme in terms of diabetic infectious wound regeneration is demonstrated, which works by glutathione peroxidase (GP*x*)‐, peroxidase (POD)‐, and catalase (CAT)‐mimetic activity for robust anti‐pathogenic property with the assistance of near‐infrared light as well as H_2_‐evolving anti‐inflammation upon solar light. A programmed‐light induced Bio‐HJzyme system for diabetic cutaneous regeneration is achieved.

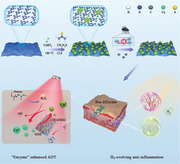

## 1 Introduction

Diabetes featuring hyperglycemia (high blood sugar) is a pressing global public health issue^[^ ^1^ ^]^ and is expected to affect over 700 million patients by 2045 (7.8% of the global population).^[^ ^2^ ^]^ The common systemic complications such as ulcer, gangrene, local infection, and diabetic foot are the main cause of death and disability for diabetic patients. A reason of such complications is the inflammatory micromilieu caused by the hyperglycemia. The high sugar level associated with such micromilieu supplies nutrition and serves as hotbed for pathogens growth; hence, further deteriorating the inflammation.^[^ ^3^ ^]^ A facile and highly effective anti‐pathogenic strategy is crucial for treating diabetic skin infection (DSI) and promoting skin regeneration.

To combat bacterial resistance in skin infection, many strategies have been proposed to disturb the normal redox reaction of bacteria, such as consuming glutathione (GSH) and enhancing reactive oxide species (ROS). Antibacterial dynamic therapy (ADT), including photodynamic therapy (PDT) and chemodynamic therapy (CDT), relies heavily on the presence of ROS.^[^ ^4^ ^]^ In recent years, nanomaterials exhibiting activities mimicking enzymes, such as catalase (CAT), peroxidase (POD), glutathione peroxidase (GP*x*), and superoxide dismutase (SOD), have been coupled with antibacterial dynamic therapy (ADT) for anti‐pathogenic applications. However, the dose of ROS generated in these therapies is often limited due to the low local production of hydrogen peroxide (H_2_O_2_) and oxygen (O_2_), as well as the insufficient separation efficiency of photoelectrons and vacancies in materials for converting H_2_O_2_/O_2_ to ROS conversion.

To overcome the aforementioned challenge, the use of glucose oxidase (GO*x*) can be a potential solution. GO*x* can reduce the in situ hyperglycemia in the diabetic microenvironment and catalyze glucose to generate abundant H_2_O_2_;^[^ ^5^ ^]^ hence, supplying a high dose of ROS required for effective ADT. On the other hand, interfacial band engineering, such as generating heterojunctions, is an promising method for photo‐induced electron–hole pairs separation via band regulation and increasing the ROS yield.^[^ ^6^ ^]^ Recent research showed that the application of heterojunctions in biomaterials (bio‐heterojunctions, bio‐HJ) offers effective remedy for pathogens‐induced diabetic wounds.^[^ ^7^ ^]^ Graphite‐phase carbon nitride (CN), a 2D nanosheet, has received wide attention in recent years and become a hotspot in the fields of photocatalysis and biomedicine.^[^ ^8^ ^]^ However, the application of CN is limited because of its high photo‐generated electron–hole pairs recombination rate and low ROS production efficiency.^[^ ^9^ ^]^ Non‐toxic copper sulfide (CuS) possesses apt bandgap energy values of ≈1.2–1.5 eV^[^ ^10^ ^]^ and favorable pathogens‐killing potency. It can be deployed to create bio‐HJ with CN for enhanced separation efficiency of photoelectrons and vacancies.^[^ ^11^ ^]^ Moreover, CN as a commonly used electrocatalytic material, can split water to form hydrogen (H_2_) under solar light, and it is well accepted that H_2_ is an emerging therapeutic gas to ameliorate inflammation by reacting with excessive ROS caused by endogenous H_2_O_2_.^[^ ^12^ ^]^

Here, we propose a revolutionary DSI treatment platform technology that deploys a programmed‐light induced H_2_‐evolving bio‐HJ enzyme (Bio‐HJzyme) composed of bio‐HJ (CN/Cu_2−*x*_S) and GO*x* with enzyme (GP*x*‐, POD‐ and CAT‐) mimicking activities. Through programmable light irradiation (near infrared [NIR] laser followed by solar light), the GO*x* decorated CN/Cu_2−*x*_S (CN/Cu_2−*x*_S@GOx) presents remarkable ADT therapy boosted by enzyme‐mimetic activities and strong anti‐inflammation effect induced by H_2_ evolution. The GP*x*‐mimetic activity of Bio‐HJzyme facilitates H_2_O_2_ production as GO*x* depleting glucose in diabetic micromilieu. The H_2_O_2_ is subsequently catalyzed into •OH through POD‐mimetic activity as well as into H_2_O and O_2_ through CAT‐mimetic activity. The H_2_O and O_2_ can be subsequently catalyzed to highly germicidal •OH and •O_2_^−^ upon NIR light irradiation. The Bio‐HJzyme exhibits integrative enzyme‐mimetic effects for enhanced ADT. Under solar irradiation (simulated by Xe light), the Bio‐HJzyme enables remarkable H_2_ production, which possesses great anti‐inflammatory property by resisting the excess ROS to safeguard surrounding cells. This work highlights the great prospects and the concept of Bio‐HJzyme triggered by programmed‐light for the treatment of deep‐seated diabetic skin infection and promotion of skin regeneration.

## 2 Results and Discussion

### 2.1 Preparation and Characterization of CN/Cu_2−*x*_S@GO*x* Bio‐HJzyme

**Scheme 1:**
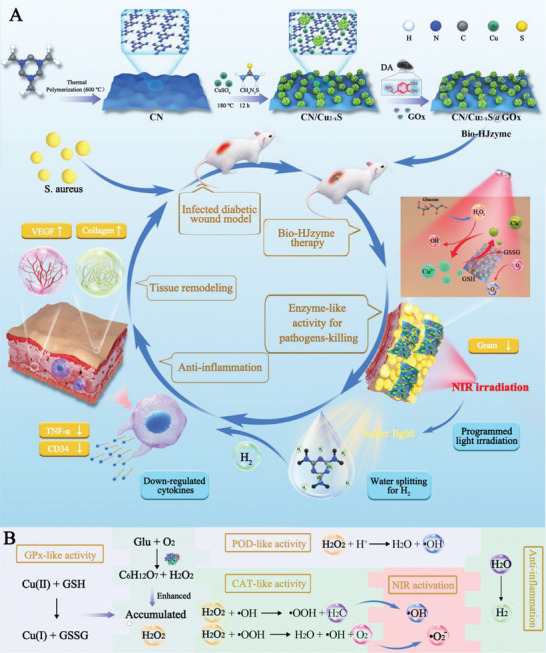
A) Schematic illustration and process for diabetic cutaneous regeneration. B) Summarization and mechanism of pathogens‐killing and anti‐inflammatory progress.

**Figure 1:**
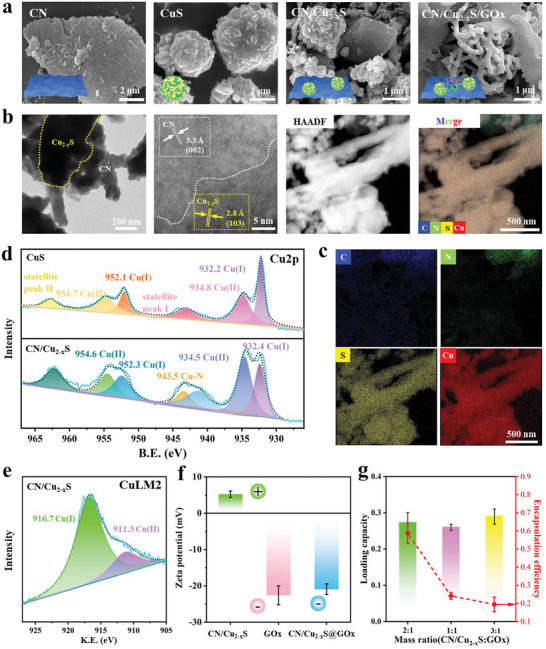
The material characterization of CN, CuS, CN/Cu_2−*x*_S, and CN/Cu_2−*x*_S@GO*x*. a) SEM images of different samples. b) The TEM image, HRTEM image, HAADF‐STEM image, and merged EDS mapping of CN/Cu_2−*x*_S. c) EDS mapping of CN/Cu_2−*x*_S for independent elements. d) The deconvolution of Cu2p in XPS analysis. e) Cu LM2 Auger peaks in XPS analysis. f) Zeta‐potential of CN/Cu_2−*x*_S, GO*x*, and CN/Cu_2−*x*_S@GO*x*. g) The GO*x* loading capacity and encapsulation efficiency on CN/Cu_2−*x*_S with varied GO*x* concentration (one‐way ANOVA and Tukey's post hoc test, *n* = 3).

The preparation of CN/Cu_2−*x*_S@GO*x* Bio‐HJzyme and the process of diabetic skin regeneration are shown in **Scheme** **1**A. Upon programmed light irradiation (NIR light followed by solar light irradiation), the Bio‐HJzyme exhibits robust anti‐pathogenic property due to the abundant ROS and follows H_2_‐evolving anti‐inflammation effect with regard to diabetic cutaneous regeneration (Scheme 1B). The SEM images of CN, CuS, CN/Cu_2−*x*_S, and CN/Cu_2−*x*_S@GO*x* are shown in **Figure** **1**a. CN are decorated by the flower‐like CuS (≈0.5–5 µm). The image of CN/Cu_2−*x*_S confirms CuS are successfully anchored to the surface of CN nanosheets via hydrothermal process. Moreover, it can be seen in CN/Cu_2−*x*_S@GO*x* that the GO*x* with wormlike structure is loaded on CN/Cu_2−*x*_S.

XRD spectra in Figure S1, Supporting Information confirm the composition and crystal structure of all samples. For CN, the stronger diffraction peaks at 27.8° and 12.8° correspond to the (002) and (100) crystal plane (JCPDS no. 87–1526). The (002) plane with 0.33 nm lattice spacing is typical of its graphite‐like structure, while the (100) plane with 0.68 nm crystal spacing reveals its interconnected tri‐s‐triazine ring.^[^ ^13^ ^]^ With regards to CuS, the diffraction peaks at 29.2°, 32.9°, and 47.9° can be ascribed to the crystal planes of (102), (006), and (110), which are consistent with the structure of hexagonal CuS (JCPDS No. 06–0464). In CN/Cu_2−*x*_S, the diffraction peaks originating from CN at 27.8° can be clearly identified, while the peak of CN at 12.8° disappears, suggesting that the CN and CuS is bounded by the tri‐s‐triazine structural units forming CN/Cu_2−*x*_S conjunction. In addition, the increased Cu_2−*x*_S intensity and decreased CN intensity are seen with increasing CuS content. Photoluminescence spectroscopy (PL) spectra, commonly used to characterize the optoelectronic properties of samples, can represent the recombination and separation rate of photocarriers. Figure S2, Supporting Information, shows the PL spectra of CN irradiated by 325 nm light. It can be seen that the strong emission peak at 460 nm is due to the recombination of photoelectron–hole pairs in CN, whereas the emission peak of CN/Cu_2−*x*_S shows a decreasing trend, indicating that the CuS can promote electron transfer and separation at the CN/Cu_2−*x*_S interface and further improve the photocatalytic efficiency. The photoelectron–hole separation efficiency follows the order: CN < CN/Cu_2−*x*_S0.1 < CN/Cu_2−*x*_S0.2 < CN/Cu_2−*x*_S0.4 < CuS < CN/Cu_2−*x*_S0.3. The results suggest low CuS content cannot provide sufficient receptors for photoinduced electrons, while abundant CuS will become the new sites for electron–hole pair recombination. The CN/Cu_2−*x*_S0.3 (abbreviated as CN/Cu_2−*x*_S) was therefore selected for further experiment. TEM images (Figure 1b) and EDS mapping (Figure 1c) demonstrate that the CuS and CN are closely combined. The crystal lattice fringes in HR‐TEM with spacing of 0.28 and 0.33 nm correspond to (103) plane in CuS and (002) plane in CN, respectively. The EDS mapping shows uniform distribution of CuS on CN. Those results prove the CN substrates provide abundant binding sites for CuS.

In order to develop an insight into the binding mechanism between Cu_2−*x*_S and CN, the chemical constitution and element states of CN, Cu_2−*x*_S, and CN/Cu_2−*x*_S were analyzed through X‐ray photoelectron spectroscopy (XPS). The C1s deconvolution of CN and CN/Cu_2−*x*_S is shown in Figure S3, Supporting Information. The peak at 288.1 eV for CN can be attributed to the N=C—N bond, while this characteristic peak increases to 288.5 eV for CN/Cu_2−*x*_S, suggesting a change of state in C. In N1s deconvolution (Figure S4, Supporting Information), the peak of CN at 398.9, 400.4, and 401.3 eV can be attributed to C—N=C, N—(C)_3_, and C—NH, respectively, while a new signal can be found for CN/Cu_2−*x*_S at 398.6 eV attributed to the C—N—Cu, indicating the chemical interaction of Cu—N between CuS and CN.^[^ ^14^ ^]^ Figure S5, Supporting Information shows the binding energy of S_3/2_ and S_1/2_ of CN/Cu_2−*x*_S is lower than that of CuS, suggesting the formation of CN/Cu_2−*x*_S bio‐HJ instead of physical mixture of CN and CuS.^[^ ^7a^ ^]^ The Cu2p deconvolution (Figure 1d) shows that both Cu(І) and Cu(II) can be found in the CuS and CN/Cu_2−*x*_S. Intriguingly, a same new signal representing Cu—N can be deconvoluted in CN/Cu_2−*x*_S group. Further, the Cu LM2 Auger peak (Figure 1e) was detected in order to determine the chemical valence state of Cu. The peaks at 916.7 and 911.3 eV can be assigned to Cu(II) and Cu(I), demonstrating the monovalent ion state of Cu. The zeta potentials of CN/Cu_2−*x*_S, GO*x*, and CN/Cu_2−*x*_S@GO*x* (Figure 1f) are —22.60 ± 2.61 mV, 5.23 ± 0.87 mV, and —20.93 ± 1.46 mV, respectively, which suggest the GO*x* were bonded to CN/Cu_2−*x*_S by electrostatic interaction.^[^ ^15^ ^]^ As shown in Figure 1g, the loading capacity of GO*x* is increased with increasing GO*x* concentration; the group with 2:1 (CN/Cu_2−*x*_S:GOx) was chosen for further in vitro and in vivo experiment.^[^ ^16^ ^]^ The gradual increased Cu release from CN/Cu_2−*x*_S@GOx in phosphate buffer (pH = 7.4) was due to the enzyme‐mimetic activity illustrated by Figure S6, Supporting Information.

### 2.2 Photothermal Properties

**Figure 2:**
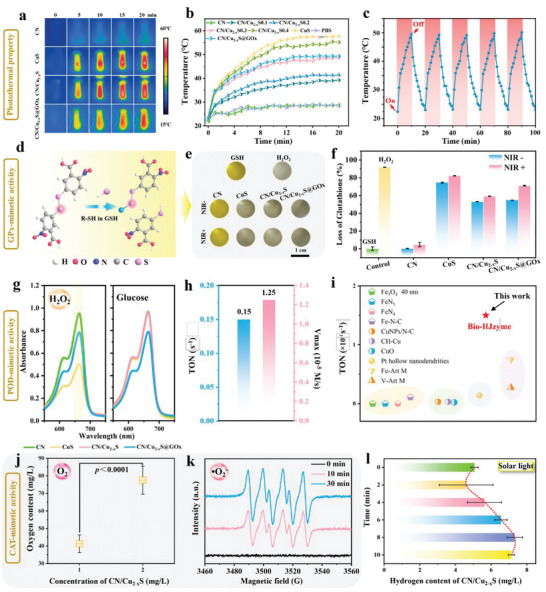
Photothermal and enzyme‐mimetic property. a) Real‐time thermal images and b) photothermal curves of various samples under 20 min NIR irradiation. c) Thermal cycle profiles of CN/Cu_2−*x*_S@GO*x* irradiated by five on/off cycles. d) Schematic demonstration of chemical reaction in DTNB oxidation and e) its corresponding color change photos as well as f) quantitative analysis demonstrated the GP*x*‐mimetic activity with and without NIR (one‐way ANOVA and Tukey's post hoc test, *n* = 3). g) UV–vis absorption spectra of MB reduced by free radicals in H_2_O_2_ and glucose, respectively. h) The calculated *V*_max_ and TON of Bio‐HJzyme. i) The comparable value of TON with other POD‐mimetic system (see references in Table S1, Supporting Information). j) The content of O_2_ catalyzed by Bio‐HJzyme (2 mg mL^−1^) in the environment of H_2_O_2_ with regards to CAT‐mimetic activity (one‐way ANOVA and Tukey's post hoc test, *n* = 3, *p* < 0.0001). k) The ESR spectra for detecting •O_2_^−^. l) The content of H_2_ for Bio‐HJzyme (2 mg mL^−1^) under solar light (one‐way ANOVA and Tukey's post hoc test, *n* = 5).

Photothermal efficiency of samples (200 µg mL^—1^) is evaluated in immersed condition (PBS). The real‐time thermal images (**Figure** **2**a) are captured under 808 nm NIR (1.5 W cm^—2^). It can be seen that all the samples present favorable photothermal ability except CN. Figure 2b shows that the CN/Cu_2−*x*_S0.3 with a temperature plateau of 48.6 °C reaches the therapeutic window (≈48–50 °C) required for bacterial inhibition.^[^ ^17^ ^]^ The temperature plateau of CN/Cu_2−*x*_S@GO*x* can reach 49.2 °C. Notably, no difference is found in the samples’ photothermal response after five heating and cooling cycles (Figure 2c), suggesting the thermal stability of the CN/Cu_2−*x*_S@GO*x*.

### 2.3 Enzyme‐Mimetic Activities for ROS Production

Bacteria maintain pivotal redox reaction by the counterbalanced ROS and glutathione (GSH) in normal physiological environment.^[^ ^18^ ^]^ DNTB was applied to detect GSH consumption according to Ellman's method.^[^ ^19^ ^]^ 5‐thio‐2‐nitrobenzoicacid (TNB), the reaction product of DTNB and sulfhydryl group (—SH) in GSH, has strong absorption at the wavenumber of 410 nm (see mechanism in Figure 2d). The color change observed in the photo (Figure 2e) and the corresponding quantitative analysis of GSH consumption (Figure 2f) reveal that without NIR irradiation, Cu_2−*x*_S, CN/Cu_2−*x*_S, and CN/Cu_2−*x*_S@GO*x* caused GSH consumption of 74.4 ± 0.50%, 53.10 ± 0.08%, and 54.69 ± 0.17%, respectively. Upon NIR irradiation, GSH consumption increased to 82.25 ± 0.25%, 59.18 ± 0.21%, and 71.14 ± 0.42%, respectively. The results suggest that CN/Cu_2−*x*_S and CN/Cu_2−*x*_S@GO*x* possess glutathione peroxidase (GP*x*)‐mimetic activity owing to the redox process of Cu(І)/Cu(II) in CN/Cu_2−*x*_S by Cu(II)+GSH → Cu(І)+GSSG, which can be further enhanced by NIR irradiation. This enzyme‐mimetic catalyst can damage the antioxidant defense system of bacteria by consuming GSH and accumulating H_2_O_2_ to effectively kill pathogens.

To trap oxidative free radicals in the presence of H_2_O_2_, we utilized methylene blue (MB) as the trapping agent, which reacts with the free radicals to result in a decreased absorption intensity. In this study, the free radical is mainly •OH. The mechanism of this process is depicted in Figure S7a, Supporting Information. The quantitative absorption intensity in the circumstances of H_2_O_2_ and glucose, respectively are shown in Figure 2g. It is found that the intensity in H_2_O_2_ follows the trend: CN > CN/Cu_2−*x*_S@GO*x* and CN/Cu_2−*x*_S > CuS, suggesting that CN/Cu_2−*x*_S has better capability of MB oxidation with a POD‐mimetic activity according to H^+^+H_2_O_2_ → H_2_O+•OH.^[^ ^20^ ^]^ In the presence of glucose, the CN/Cu_2−*x*_S@GO*x* group shows sharp decrease for absorption intensity compared with the other groups owing to the presence of H_2_O_2_ produced by GO*x* following: C_6_H_12_O_6_ + O_2_ + H_2_O → C_6_H_12_O_7_ + H_2_O_2_. Furthermore, the process can be strengthened under NIR irradiation (Figure S7b, Supporting Information). Michaelis–Menten steady‐state kinetics was applied to evaluate the catalytic efficiency of our Bio‐HJzyme. The absorbance change of catalytic reaction was monitored to obtain the kinetic data, where higher H_2_O_2_ concentration induced greater •OH generation. As shown in Figure 2h, the maximum initial velocity (*V*_max_) and turnover number (TON) were 1.25 × 10^−5^ m s^−1^ and 0.15 s^−1^, respectively. To compare the POD‐mimetic effect of Bio‐HJzyme with other systems reported by the literature (see references in Table S1, Supporting Information), we performed the TON of this study and several other POD‐mimetic nanozymes in Figure 2i. In comparison, Bio‐HJzyme demonstrated remarkably higher catalytic efficiency compared to Fe‐based and other Cu‐based nanozymes. The enhanced efficiency was attributed to the unique structure of bHJ, which effectively separated photoelectrons and vacancies.

To assess the CAT‐mimetic activity of CN/Cu_2−*x*_S@GO*x* in the presence of H_2_O_2_, the amount of catalyzed O_2_ was measured and plotted in Figure 2j. Results indicate that the O_2_ content produced by 1 and 2 mg/mL^−1^ CN/Cu_2−*x*_S@GO*x* were 41.3 and 77.5 mg L^−1^, respectively, which demonstrates the high efficiency of the Bio‐HJzyme to catalyze H_2_O_2_ into O_2_. Electron spin resonance (ESR) spectrometer was utilized to identify the types of ROS. The characteristic signal peaks of the DMPO‐•OH (Figure S8, Supporting Information) and DMPO‐•O_2_^−^ (Figure 2k) adducts were detected with and without NIR irradiation. The results reveal that the CN/Cu_2−*x*_S can produce •OH and •O_2_^−^ under NIR irradiation by catalyzing H_2_O and O_2_, respectively.

H_2_ therapy is considered an emerging anti‐inflammatory strategy via suppressed oxidative stress in local physiological environment.^[^ ^12b^ ^]^ Thus, the H_2_ produced by CN/Cu_2−*x*_S@GOx group under solar light irradiation (Xe lamp) is carried out. Figure 2l shows H_2_ content increases over time, and it can react with the excess ROS produced under NIR irradiation to form water.

### 2.4 Density Functional Theory Calculations

**Figure 3:**
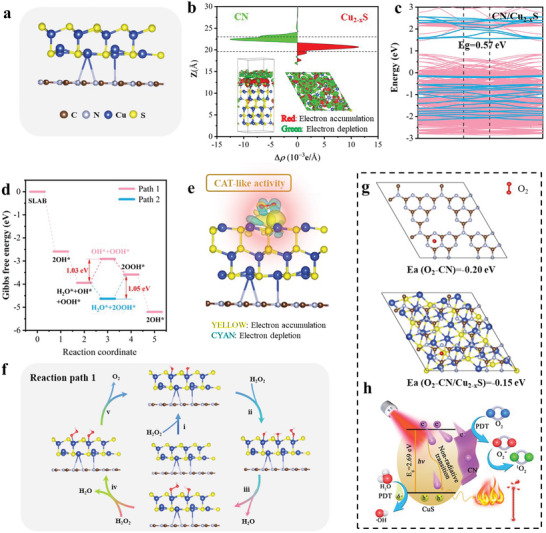
DFT calculations. a) Side views of CN/Cu_2−*x*_S structure. b) The planar‐averaged charge density difference (2D) of CN/Cu_2−*x*_S; inset is the top and side views of the charge density difference (3D) of CN/Cu_2−*x*_S. c) Calculated electronic band structures of unit cell of CN/Cu_2−*x*_S. d) Gibbs free energy of potential pathways on the catalysis of H_2_O_2_. e) Charge density difference at Cu of path 1 (yellow and cyan represent charge accumulation and depletion, respectively). f) Proposed reaction pathways of H_2_O_2_ catalysis. g) The CN and CN/Cu_2−*x*_S atomic model after O_2_ absorption, respectively. h) Schematic illustration of the mechanism of photothermal and NIR enhanced CAT‐mimetic properties in CN/Cu_2−*x*_S@GO*x* group under NIR laser irradiation.

Density functional theory (DFT) calculations are carried out to demonstrate the interfacial interaction between CN and CuS. **Figure** **3**a shows the bio‐heterojunction structure of CN/Cu_2−*x*_S. The charge density difference in CN/Cu_2−*x*_S (Figure 3b) shows that electrons are concentrated at the heterointerface. The abundance of electrons at the top of CuS and the electron‐deficient region at the bottom of CN provide strong evidence that electrons can transfer from CuS to CN. This transfer behavior is efficiently regulated by CN/Cu_2−*x*_S through the redistribution of electrons at the interface, as evidenced by the planar‐averaged differential charge density Δ*ρ* along the *Z* direction. To better demonstrate this regulatory effect, the contributions of CuS to the electronic band gap of CN are simulated; see Figure 3c; Figure S9, Supporting Information. The calculated energy bands of CN, CuS, and CN/Cu_2−*x*_S are 1.19, 1.11, and 0.57 eV, respectively. The results show that the introduced CuS induces the formation of impurity levels in the band gap of CN; thus, promoting the electrons generation and reducing the band gap of CN. The reduced band gap is beneficial to produce more photogenerated electrons under the NIR irradiation; thus, accelerating the ROS yields. Figure S10a, Supporting Information shows the valence bands (VB) of CN and CuS calculated by experimental XPS analysis, which are 1.65 and 1.07 eV, respectively. The different value between experiment and DFT calculations can be attributed to the generalized gradient approximation (GGA) Perdew–Burke–Emzerh (PBE) equation properties.^[^ ^21^ ^]^ Moreover, the bandgaps of CN and CuS (fitted by tauc plot method according to UV–vis–NIR spectra [Figure S10b, Supporting Information]) are 2.69 and 1.66 eV, respectively. The conduction bands (CB) of CN and CuS are determined to be −1.04 and −0.59 eV, respectively (vs NHE).

Furthermore, the calculation of CAT‐mimetic pathways has revealed that the H_2_O produced during the reaction process was strongly adsorbed on the CN/Cu_2−*x*_S catalyst due to its electron enrichment, making desorption difficult. Two potential CAT‐mimetic paths were proposed and the Gibbs free energy of all intermediates in each reaction step were calculated. Figure 3d shows the H_2_O desorption was regarded as the rate‐determining step in the proposed paths, in which path 1 showed a lower energy barrier (1.03 eV) than path 2 (1.05 eV).^[^ ^22^ ^]^ In addition, the differential charge density of H_2_O_2_ was analyzed (Figure 3e), which shows that most electrons on the active site were concentrated at H_2_O_2_ molecule and the Cu atom. Figure 3f shows the crucial Gibbs free energy and intermediate structures diagrams along the optimized reaction paths during the CAT‐mimetic catalytic H_2_O_2_ decomposition. First, the H_2_O_2_ molecule was adsorbed on a Cu site and rapidly dissociated into two OH* species. Second, one of the OH* species reacted with another H_2_O_2_ molecule, producing a •OOH species and a H_2_O* molecule. The H_2_O* molecule then desorbed from the surface. Following this, the other OH* reacted with the third H_2_O_2_ to form two OOH*. Thus, two OOH* reacted with each other and generated O_2_ and H_2_O_2_, and finally, H_2_O_2_ was re‐decomposed into two OH* and started the next catalytic cycle.^[^ ^20^ ^]^

The production of ROS, such as •O^2−^ and ^1^O_2_ in PDT, required the O_2_ participation, including the processes of adsorption and dissociation. The O_2_ adsorption efficiency was primary because O_2_ could react with photogenerated electrons and subsequently affect the ROS generation. DFT calculation was employed to offer new insights into the photocatalysis process of O_2_ adsorption on CN and CN/Cu_2−*x*_S (Figure 3g). The results show that the active site of O_2_ adsorption can be found in both CN and CN/Cu_2−*x*_S. To further explore this feature, the respective O_2_ adsorption energy was calculated. The lower adsorption energy indicated a better absorption efficiency of O_2_. Compared with CN (−0.2 eV), CN/Cu_2−*x*_S demonstrated lower adsorption energies (−0.15 eV), which improved the adsorption of O_2_ and promoted the photodynamic process more effectively.

From the DFT calculations and experimental results, the mechanisms of the photothermal and NIR enhanced CAT‐mimetic properties of the CN/Cu_2−*x*_S are proposed in Figure 3h. Under NIR irradiation, the electrons in the CB of CN (−1.04 eV) can easily transfer to the CB of CuS (−0.59 eV) owing to the closer CB compared with the VB of CN. This effectively reduces the rapid recombination of light‐excited electron–hole pairs in CuS and CN, respectively.^[^ ^23^ ^]^ Moreover, the standard reduction potentials of (O_2_/•O_2_^−^) are −0.33 eV,^[^ ^24^ ^]^ which are more positive than the CB of CuS (−0.59 eV), indicating that the CN/Cu_2−*x*_S system can generate •O_2_^−^ under NIR irradiation by consuming O_2_. These results indicate that CN/Cu_2−*x*_S has a great potential to speed up ROS production, especially •O_2_^−^.

### 2.5 In Vitro Antibacterial Property

**Figure 4:**
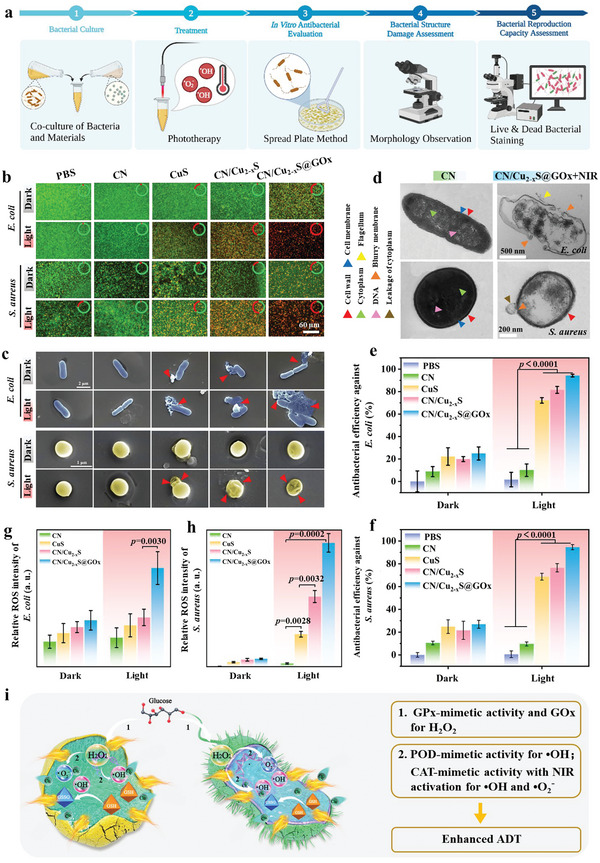
In vitro antibacterial test. a) The schematic antibacterial process. b) Live/Dead (green/red) fluorescence images of *E. coli* and *S. aureus* with/without NIR laser illumination. c) SEM images of bacterial changes of *E. coli* and *S. aureus* with/without NIR laser illumination (red arrows: disrupted bacterial membrane). d) TEM images of *E. coli* and *S. aureus* morphologies treated with CN and CN/Cu_2−*x*_S@GO*x* with 808 nm NIR laser. Antibacterial efficiency against e) *E. coli* and f) *S. aureus* with/without NIR irradiation, respectively (one‐way ANOVA and Tukey's post hoc test, *n* = 5, *p* < 0.0001). Semi‐quantitative analysis of intracellular ROS intensity of g) *E. coli* and h) *S. aureus* detected by DCFH‐DA (one‐way ANOVA and Tukey's post hoc test, *n* = 5, *p* < 0.01). i) Anti‐pathogens mechanism.

The photoinduced capability of the samples has motivated further investigation into their antibacterial properties; for results, see **Figure** **4**a. To gain deeper insight into the bactericidal behavior of the samples, Live/Dead staining, SEM observation, and TEM observation are performed. Live/Dead staining (Figure 4b) reveals that the groups treated with CuS + NIR, CN/Cu_2−*x*_S + NIR, CN/Cu_2−*x*_S@GO*x*, and CN/Cu_2−*x*_S@GO*x* + NIR produce amplified red fluorescence, indicating enhanced antibacterial effects. GO*x* is found to produce more H_2_O_2_, while CN/Cu_2−*x*_S converts H_2_O_2_ into •OH and •O_2_^−^ radicals. Bio‐HJzyme shows further enhanced antibacterial properties under NIR irradiation. SEM imaging (Figure 4c) shows that bacteria cultured with CN have intact cytoplasmic membranes with rod or spherical morphology, indicating weak anti‐pathogen effects. In contrast, CuS, CN/Cu_2−*x*_S, and CN/Cu_2−*x*_S@GO*x* with/without NIR irradiation cause partial shrinkage/distortion of the cytoplasmic membrane and cytoplasm leakage due to membrane breakage. TEM images (Figure 4d) show that bacteria co‐cultured with CN/Cu_2−*x*_S@GO*x* with NIR irradiation have deformed morphology, blurry membranes, and leaked intracellular substrates due to induced local hyperthermia and produced ROS, which damage the phospholipid bilayers of the bacterial membrane, disturb bacterial membrane permeability, and ultimately, cause bacterial matrix leakage.

The spread plate method was used to quantify the number of bacterial colonies for different groups.^[^ ^25^ ^]^ The results (Figure S11, Supporting Information) show a slight decrease in bacterial colonies for CuS, CN/Cu_2−*x*_S, and CN/Cu_2−*x*_S@GO*x* groups compared to PBS and CN groups. However, the bacterial colonies further decrease under NIR laser irradiation, indicating the remarkable antibacterial effect activated by NIR laser. A similar trend is also found on *Staphylococcus aureus*. The quantitative analysis against *Escherichia coli* is summarized in Figure 4e, which demonstrates that the antibacterial efficiency of CN against *E. coli* with and without NIR is 8.76% ± 4.48% and 9.97% ± 5.54%, respectively, implying NIR irradiation has no obvious effects on the antibacterial efficiency of CN owing to its low photothermal effect. The antibacterial efficiencies of CuS, CN/Cu_2−*x*_S, and CN/Cu_2−*x*_S@GO*x* are 22.12% ± 7.77%, 19.91% ± 2.22%, and 24.86% ± 5.83% respectively, which can be substantially increased to 72.16% ± 2.47%, 81.50% ± 3.09%, and 94.22% ± 1.25% with the excellent NIR‐triggered therapy, indicating the remarkable antibacterial effect caused by the NIR laser activation. There is a similar trend against *S. aureus* as shown in Figure 4f. The antibacterial efficiencies of CuS, CN/Cu_2−*x*_S, and CN/Cu_2−*x*_S@GO*x* are 24.79% ± 6.00%, 21.52% ± 7.99%, and 26.83% ± 3.50% respectively, which can reach 68.60% ± 2.98%, 76.46% ± 3.64%, and 94.53% ± 2.26% under NIR illumination, implying the favorable bacterial inhibition of CN/Cu_2−*x*_S@GO*x* caused by the above‐mentioned synergistic effect. Furthermore, the 2′,7′‐Dichlorodihydrofluorescein diacetate (DCFH‐DA) assay is applied for detecting the intracellular ROS formation in different groups. Figure S12, Supporting Information, shows the green fluorescence representing intracellular ROS intensity of bacteria is significantly enhanced for CN/Cu_2−*x*_S and CN/Cu_2−*x*_S@GO*x* with NIR irradiation owing to the robust separation efficiency of electron–hole pair originating from the unique structure of bHJ. The corresponding semi‐quantitative analyses of ROS intensity for *E. coli* and *S. aureus* are displayed in Figure 4g,h, which display that there is significant difference between CN + NIR and CN/Cu_2−*x*_S@GO*x* + NIR, indicating the Bio‐HJzyme facilitates ROS production for strong anti‐pathogenic effect.

All the above results show that the structure and properties of CN/Cu_2−*x*_S@GO*x* can effectively strengthen separation efficiency of electron–hole pair and enhance the ROS yield to attain the effective sterilization under NIR irradiation. The associated mechanism is shown in Figure 4i. 1) The GSH consumption works by cyclic Cu(II)/Cu(І) (GP*x*‐mimetic activity) bringing about H_2_O_2_ accumulation, which can be amplified with GO*x*; 2) POD‐mimetic activity of Bio‐HJzyme catalyzes H_2_O_2_ to germicidal •OH. At the same time, the H_2_O_2_ can be catalyzed to H_2_O and O_2_ (CAT‐mimetic activity), which can be further catalyzed to •OH and •O_2_^−^ under NIR irradiation.

### 2.6 In Vitro Cytocompatibility

The cytocompatibilities of CN, CuS, CN/Cu_2−*x*_S, and CN/Cu_2−*x*_S@GO*x* cultured with L929 were evaluated using Live/Dead staining and CCK‐8 assays. Live/Dead staining (Figure S13, Supporting Information) reveals that the cells with CN group represent green fluorescence (indicating live cells), suggesting the favorable cytocompatibility of CN. In contrast, the CuS, CN/Cu_2−*x*_S, and CN/Cu_2−*x*_S@GO*x* groups exhibit a proportion of red fluorescence caused by Cu(II), •OH, and •O_2_^−^ radicals generated by CN/Cu_2−*x*_S@GO*x*. The quantitative CCK‐8 results (Figure S14, Supporting Information) show that the cell viabilities of CuS, CN/Cu_2−*x*_S, and CN/Cu_2−*x*_S@GO*x* groups are weaker than those of the CN group. However, with increased culture time, all groups show steady growth, implying that CN/Cu_2−*x*_S@GO*x* has better cytocompatibility for diabetic skin repair.

### 2.7 In Vivo Diabetic Skin Repair

**Figure 5:**
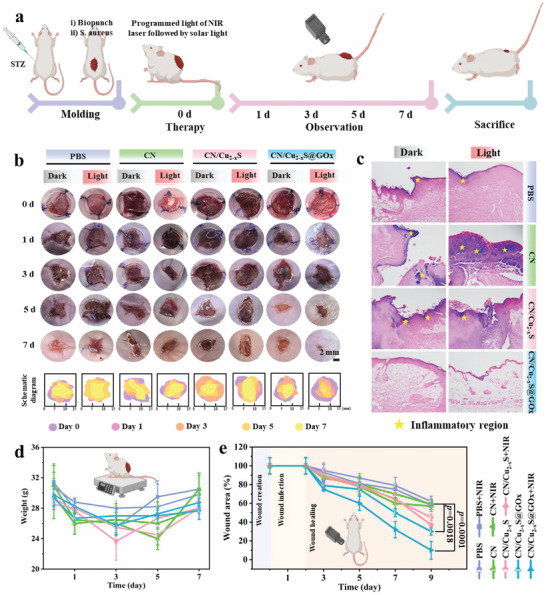
In vivo antibacterial evaluation around implants in diabetic infected rat model. a) Schematic illustration of in vivo experimental procedure. b) The wound photos and schematic diagram of diabetic mice skin on days 0, 1, 3, 5, and 7. c) Immunohistochemistry images of Gram staining; the yellow star indicates the inflammatory region. d) The body weight of diabetic mice during treatment (one‐way ANOVA and Tukey's post hoc test, *n* = 5). e) The change curves of wound area calculated by wound photos (one‐way ANOVA and Tukey's post hoc test, *n* = 5, *p* < 0.01).

The antibacterial properties and skin repair capabilities of the different samples were further evaluated using a diabetic mouse model; for details, see **Figure** **5**a. The infrared images of the samples applied on diabetic mice skin are displayed in Figure S15, Supporting Information. No significant temperature changes can be observed in the PBS and CN groups under NIR irradiation, while a favorable photothermal effect is observed in the CN/Cu_2−*x*_S and CN/Cu_2−*x*_S@GO*x* groups. The corresponding temperature curves are displayed in Figure S16, Supporting Information. Within 3 min, the temperatures of CN/Cu_2−*x*_S and CN/Cu_2−*x*_S@GO*x* can reach to 45 °C and 48 °C, respectively. The increase in temperature can enhance the bacterial sensitivity to the stimulation of ROS, leading to enhanced sterilization.^[^ ^7a^ ^]^

The wound in diabetic individuals is characterized by inflammation and suppuration, which is worsened by bacterial infection. To investigate the wound healing potentials of CN, CuS, CN/Cu_2−*x*_S, and CN/Cu_2−*x*_S@GO*x* with and without NIR treatment, photos of wound alteration are shown in Figure 5b. All groups exhibit a decrease in wound area over time, but CN/Cu_2−*x*_S@GO*x* with NIR irradiation shows the best wound healing effect with the minimum wound area on day 7. In contrast, other groups show obvious crust and are not fully healed. Gram staining is performed to detect the intensity of bacterial infection after treatment. Figure 5c reveals that CN/Cu_2−*x*_S@GO*x* + NIR group displays the least amount of violet stain, indicating a favorable effect on bacterial inhibition during the diabetic skin repair.

To determine the impact of treatment on the health of diabetic mice, body weight of the mice is measured for 7 days. As shown in Figure 5d, no significant negative effects are observed for CN/Cu_2−*x*_S@GO*x* and NIR treatment, suggesting this is a benign therapy. The quantitative analysis of diabetic wound area is presented in Figure 5e. The trend of wound area follows: CN/Cu_2−*x*_S@GO*x* + NIR < CN/Cu_2−*x*_S@GO*x* < CN/Cu_2−*x*_S + NIR < CN/Cu_2−*x*_S < CN + NIR ≈ CN ≈ PBS + NIR ≈ PBS. Notably, the wound area of CN/Cu_2−*x*_S@GO*x* group on day 7 is only 10% ± 9.57% of the original area, indicating that Bio‐HJzyme effectively kills bacteria and promotes diabetic wound skin repair with the aid of NIR irradiation.

**Figure 6:**
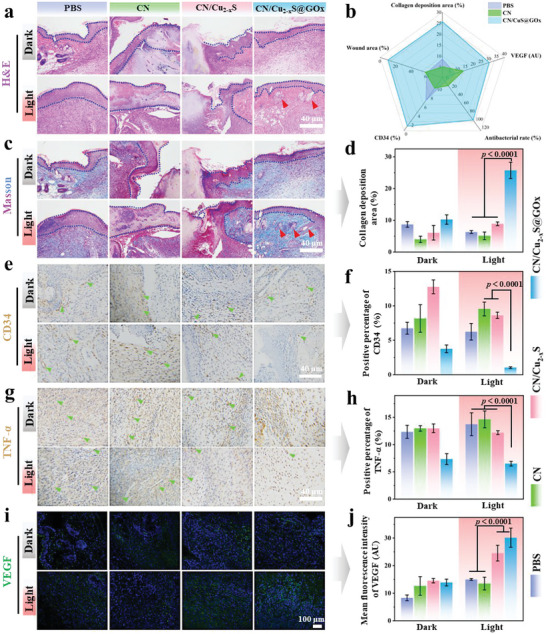
Biochemical and immunohistochemistry analysis. a) H&E staining of skin tissues. b) RadarChart to compare collage deposition area, TNF‐*α*, CD34, and antibacterial efficiency against *S. aureus* and *E. coli* in groups of CN, CN/Cu_2−*x*_S, and CN/Cu_2−*x*_S@GO*x*. Immunohistochemistry staining images and the corresponding quantitative analysis of c,d) Masson's trichrome, e,f) CD34, g,h) TNF‐*α*, and i,j) VEGF of skin tissues in mice wounds on day 7; green arrows indicate the inflammatory area (one‐way ANOVA and Tukey's post hoc test, *n* = 3, *p* < 0.0001).

To investigate the inflammation around the wound, H&E staining is applied and the results are presented in **Figure** **6**a. The groups without NIR irradiation show a high number of inflammatory cells and acute neutrophil infiltration, indicating a typical feature of soft‐tissue infection due to severe bacterial infection. However, the CN/Cu_2−*x*_S@GO*x* +NIR group exhibits few inflammatory cells, even forms a more complete epidermis, and develops hair follicles (red arrow), indicating the strong potential for diabetic skin repair. Moreover, other groups with NIR show a small amount of inflammatory cells, indicating that bacteria are significantly reduced. This validates the remarkable antibacterial capacity and anti‐inflammation of CN/Cu_2−*x*_S@GO*x* group in in vivo experiments.

A RadarChart has been constructed to compare the collage deposition area, TNF‐*α*, CD34, and antibacterial efficiency against *S. aureus* and *E. coli* in groups of CN, CN/Cu_2−*x*_S, and CN/Cu_2−*x*_S@GO*x*; see Figure 6b. The maximum area is found in CN/Cu_2−*x*_S@GO*x* group, indicating the superior property of our Bio‐HJzyme. Specifically, Masson staining is used to evaluate skin tissue regeneration by staining collagen fibers blue. The results show that the CN/Cu_2−*x*_S@GO*x* group has more blue‐stained collagen fibers that are arranged continuously, and even, develops many new hair follicles (yellow arrow) compared with other groups, implying its excellent capability for diabetic skin repair, as depicted in Figure 6c. The semi‐quantitative statistical analysis of collagen deposition area is displayed in Figure 6d. The groups of PBS and CN exhibit a similar level of collagen deposition, while the CN/Cu_2−*x*_S@GO*x* group demonstrates a higher collagen deposition, which is further enhanced with the assistance of NIR irradiation.

In skin repair, the secretion of anti‐inflammatory factors such as CD34 and TNF‐*α* by cells plays a critical role. CD34, expressed on a subset of fibrocytes, has been widely investigated in clinical trials for its potential to enhance wound healing, mainly due to its ability to stimulate angiogenesis.^[^ ^26^ ^]^ Figure 6e shows more intense CD34 staining in the CN/Cu_2−*x*_S@GO*x* + NIR group, indicating higher CD34 levels on day 7 of the diabetic wound healing process. On the other hand, TNF‐*α* is an inflammatory cytokine that can inhibit wound healing in diabetic conditions.^[^ ^27^ ^]^ The TNF‐*α* staining (Figure 6g) shows a reduction in TNF‐*α* levels with NIR irradiation, which is beneficial to diabetic wound healing. The semi‐quantitative statistical analysis of CD34 (Figure 6f) and TNF‐*α* (Figure 6h) demonstrates that the CN/Cu_2−*x*_S@GO*x* + NIR group has reduced levels of inflammatory cytokines compared to other groups, which can be attributed to the H_2_ split from water under the solar light irradiation (Xe lamp).

Vascular endothelial growth factor (VEGF) is a crucial regulator of vascular development and plays a key role in the function of blood and lymphatic vessels, ultimately accelerating wound closure and healing by stimulating angiogenesis.^[^ ^28^ ^]^ However, in diabetic patients, local production of VEGF is often limited, resulting in delayed wound healing. In Figure 6i, it can be seen that CN/Cu_2−*x*_S@GO*x* exhibits a stronger fluorescence intensity, which is further enhanced in the CN/Cu_2−*x*_S@GO*x* + NIR group. The green fluorescence is quantitatively analyzed; see Figure 6j. It is evident that the CN/Cu_2−*x*_S@GO*x* + NIR and CN/Cu_2−*x*_S + NIR groups show the highest fluorescence intensity. The immunohistochemical analysis reveals that the CN/Cu_2−*x*_S@GO*x* + NIR group possesses the optimum capability for collagen deposition, anti‐inflammation, and angiogenesis, making it an effective pathway for sterilization and skin repair in diabetic wounds.

## 3 Conclusion

Our study presents the design and development of a novel H_2_‐evolving Bio‐HJzyme consisting of CN/Cu_2−*x*_S and GO*x*, the function of which can be activated by programmable light for the purpose of diabetic infectious skin treatment and regeneration. Our findings demonstrate that the Bio‐HJzyme possesses strong anti‐infection capabilities against both Gram‐negative and Gram‐positive pathogens under NIR irradiation. This is attributed to the triple enzyme‐mimetic activity, namely GP*x*‐, POD‐, and CAT‐mimetic activity. Moreover, the in vivo animal evaluation highlights the ability of Bio‐HJzyme to evolve H_2_ under solar light, which can effectively reduce the inflammatory response caused by residual ROS, which further promotes angiogenesis and regeneration of diabetic skin tissues. This work provides a transformative approach for diabetic wounds healing which may also be generalized for other skin infection/healing applications.

## 4 Experimental Section

### Preparation of CN Powders

CN powders were synthesized by thermal polymerization. Specifically, 6 g melamine (Chron Chemicals, Chengdu, China) was calcined for 2 h at 600 °C with a heating rate of 5 °C min^−1^ under air atmosphere, which cooled naturally to the room temperature after the reaction. The yellow CN products were collected.

### Preparation of CN/Cu_2−*x*_S

CN/Cu_2−*x*_S was synthesized by hydrothermal process as described. Briefly, 1 g CN was dispersed into 100 mL deionized water with 2 h ultrasonic treatment. Copper sulfate (CuSO_4_·5H_2_O, Chron Chemicals) and thiourea (CH_4_N_2_S, Chron Chemicals) with different mass ratios of 0.1 g/0.05 g, 0.2 g/0.1 g, 0.3 g/0.15 g, and 0.4 g/0.2 g were dissolved into deionized water, respectively and mixed uniformly with previous CN powders. The solution was transferred to a hydrothermal kettle and reacted at 180 °C for 24 h. After being rinsed with ethanol and deionized water for several times, filtered and placed in a 60 °C oven for drying, the CN/Cu_2−*x*_S was named as CN/Cu_2−*x*_S0.1, CN/Cu_2−*x*_S0.2, CN/Cu_2_−*_x_* S0.3, and CN/Cu_2−*x*_S0.4, respectively.

### Preparation of CN/Cu_2−*x*_S@GO*x* Bio‐HJzyme

A 0.2 mg mL^−1^ dopamine (DA) solution was prepared by dissolving DA in Tris solution (pH = 8.4). 2 mg mL^−1^ CN/Cu_2−*x*_S was mixed with the prepared DA solution and mixed for 12 h. The resulting sample was then washed and dried before being immersed in a 2 mg mL^−1^ GO*x* solution dissolved in PBS for an additional 12 h at 4 °C. After washing and drying at 37 °C, the resulting GO*x*‐decorated CN/Cu_2−*x*_S (CN/Cu_2−*x*_S@GOx Bio‐HJzyme) was obtained.

### Materials Characterization

Scanning electron microscope (SEM, JSM‐7500F, JOEL, Japan) and transmission electron microscope (TEM, Talos F200X, Thermo scientific, US) were applied to observe the diffraction pattern, microstructures, topography, and elemental distribution of all samples. X‐ray photoelectron spectroscopy analysis (XPS, XSAM800, Kratos, England), Raman spectra (InVia, Renishaw, UK), and Fourier transform infrared spectroscopy (FTIR, Nicolet 6700, US) were conducted for the analysis of chemical bonding. The composition of the sample was detected by X‐ray diffraction analysis (XRD, XRD‐6100, Japan). Zeta potential (Zetasizer Nano ZS, Malvern, England) was used to measure the surface charge of particle. The absorption spectrums ranging from 200 to 1000 nm were detected by UV–vis spectrophotometer (UV3600, JEOL). The tauc plot method was used to fit out the band gap.^[^ ^29^ ^]^ The free radicals with and without laser irradiation were measured via ESR (Bruker A300, Germany).

### DFT Calculations

For H_2_O_2_ catalysis, all spin‐polarized density functional theory (DFT) method was performed using the Vienna ab initio simulation package (VASP) code with the projector augmented wave (PAW) method.^[^ ^30^ ^]^ The generalized gradient approximation (GGA) combined with Perdew–Burke–Ernzerhof (PBE) was employed to describe the exchange‐correlation term.^[^ ^31^ ^]^ The PAW pseudo‐potentials were used to describe ionic cores. The cut off energy for the plane‐wave basis was set to 450 eV. The van der Waals (vdW) interactions were described by using the empirical correction in Grimme's scheme (DFT‐D3) in all calculations.^[^ ^32^ ^]^ The convergence tolerances for energy and force were set to 10−5 eV and 0.05 eV Å−1, respectively. The Gibbs free energy change (Δ*G*) for each elemental step was defined as the following Equation (1):

(1) $$\Delta G=\Delta E_{DFT}+\Delta E_{ZPE}-T\Delta S$$

In this equation, Δ*E*_DFT_ denotes the electronic energy change directly obtained from DFT calculations, Δ*E*_ZPE_ is the zero‐point energy correction, and Δ*S* is the entropy change obtained from frequency calculations at 298.15 K.

For elucidating the surface electron transfer mechanism of CN and Cu_2−*x*_S, first‐principle calculations were conducted utilizing the VASP. The valence‐core electron interaction was disposed by employing PAW method, and the exchange of electron and related interaction was described through the GGA PBE function. For the long‐range interference in the CN/Cu_2−*x*_S interface, vdW interaction was deliberated based on the Grimme's DFT‐D3 correlation to attain a better depiction of intermolecular interaction whereas the dynamic energy cut‐off in the plane‐wave basis function was configured to 400 eV. Besides, the change of energy (Δ*E*) for O_2_ adsorption by CN/Cu_2−*x*_S was computed on the basis of the following Equation (2):

(2) $$\Delta E=E_{a}-E_{0}$$

where *E*_a_ is the gross energy for the O_2_ adsorbed CN/Cu_2−*x*_S and *E*_0_ is the energy of the pure surface of CN/Cu_2−*x*_S.

### Photothermal Performance

The photothermal performance was tested in PBS. A 808 nm NIR (Changchun Leishi Optoelectronic Technology co., LTD, MW‐GX‐808/2000 mW) with laser power densities of 1.5 W cm^−^^2^ was applied to prepared samples with a concentration of 1 mg mL^−1^. The temperature was recorded using thermal imaging system (TiS20+, Fluke, USA). The photothermal cycle was conducted to explore the photothermal stability of the samples. The temperature was recorded every 60 s.

### GP*x*‐Mimetic Activity

GSH consumption was detected. 50 µL sample solution (2 mg mL^−1^) and 450 µL GSH solution (1 mm), which was dissolved in carbonate buffer (pH = 8.0) were mixed in the dark for 60 min. Negative control group (500 µL GSH solution) and positive control group (450 µL GSH solution and 50 µL 0.1 m H_2_O_2_) were established. After incubation and lighting for 10 min, 450 µL TRIS‐HCl buffer (50 mm, pH = 8) and 100 µL 5,5′‐dithio‐bis‐nitrobenzoicacid (DTNB, 10 mm) were added to the mixed liquid. After 30 min, the absorbance of the supernatant was measured at 410 nm, and the full spectrum scanning at ≈250–500 nm was performed by UV–vis.

### POD‐Mimetic Activity

POD‐mimetic activities was processed by •OH detection. PL was used for detecting the separation ability of photogenerated electron‐hole pairs of the prepared samples. The MB degradation was applied to determine the generation ability of •OH by the samples with and without glucose. In brief, 200 µL H_2_O_2_ (10 mm) or glucose (5 mg mL^−1^) was first added into the 4.3 mL 20 mg mL^−1^ MB solution and incubated for 30 min in the dark. After that, differently as‐prepared solutions (700 µL, 1 mg mL^−1^) were added into the H_2_O_2_‐MB mixture and reacted for 30 min. The UV–vis absorbance was measured at the wavelength from 550 to 750 nm. The •OH production was further measured at each 5 min interval using ESR (JES‐FA200, JEOL, Japan). MB and 5, 5‐dimethyl‐1‐pyrrolidine N‐oxide (DMPO, Dojindo, Japan) were applied for trapping •OH.

### Catalytic Efficiency of POD‐Mimetic Activity

POD‐mimetic activity of Bio‐HJzyme was detected by colorimetric assays. 500 µL of samples (0.1 mg mL^−1^), 20 µL of 10 mg mL^−1^ TMB, and 25 µL of 1 m H_2_O_2_ were added into 2 mL 100 mm sodium acetate–acetic acid (NaOAc/HOAc, pH = 4.2) buffer. The catalytic oxidation of TMB was studied by detecting the absorption changes at *λ*_max_  =  652 nm. Michaelis–Menten steady‐state kinetics were used to evaluate the catalytic efficiency. *V*_max_ was calculated based on the Lineweaver–Burk plots of the double reciprocal of the Michaelis–Menten Equation (3).

(3) $$V=V_{\max}\times \left[S\right]/\left(\mathrm{Km}+\left[S\right]\right),\mathrm{TON}=V_{\max}/\left[E\right]$$

where [*S*] is the concentration of H_2_O_2_ and [*E*] is the molar concentration of metal in Bio‐HJzyme.^[^ ^33^ ^]^

### CAT‐Mimetic Activity

CAT‐mimetic activity was processed by O_2_ detection, which was tested by dissolved oxygen tester (Leici JPB‐607A, Shanghai Yiden Scientific Instrument Co., LTD). Bio‐HJzyme was immersed into 10.0 vol% H_2_O_2_ up to a stable value. ESR was also used to detect •O_2_^−^ production at each 5 min interval under NIR irradiation for the capability of turn O_2_ to •O_2_^−^.

### H_2_ Detection

The H_2_ content was detected by gas chromatograph (GC, America, Agilent 7890B). Specifically, CN/Cu_2_−*_x_* S@GO*x* was dispersed in water with a concentration of 2 mg mL^−1^, which was first degassed with Ar stream (10 mL min**^−1^**). The solution was irradiated by Xe lamp (for simulating solar light) and connected with GC for H_2_ detection.

### In Vitro Antibacterial Property

*E. coli* (ATCC25922) and *S. aureus* (ATCC25923) were used to study the antibacterial properties of the samples. The antibacterial efficiency was measured according to lab's protocol.^[^ ^15^, ^34^ ^]^ Specifically, 40 µL 200 µg mL^−1^ sample dispersion was mixed with 160 µL bacterial solution. For the NIR group, the mixed solution was treated by NIR irradiation for 10 min. Further, the morphology of bacteria was observed by SEM and Live/Dead staining (SYTO9/PI). Moreover, the bacteria treated with various samples with/without NIR (808 nm, 1.5 W cm^−2^) light were stained using a Live/Dead Bacteria Viability Kit (Thermo‐Fisher, USA) following the instruction. After staining, the samples were observed by a fluorescent inverted microscope (CKX53, Olympus, Japan) in which the live bacteria were stained to green by SYTO‐9, while dead bacteria were dyed to red by propidium iodide.

### Animal Experiment

The surgical procedures were approved by the Animal Ethics Committee of West China Hospital of Sichuan University, China (approval number: 2021605A). Mice were supplied by Chengdu Dossy Experimental Animal Co., LTD. The diabetic mouse model was achieved by streptozocin (STZ) injection to evaluate the disinfection property of samples.^[^ ^35^ ^]^ In experiment, a *Φ* 8 mm wound was created on the mice back. 20 µL *S. aureus* (1 × 10^7^ CFU per mL) was added to establish a diabetic cutaneous defect infection model.^[^ ^7b^ ^]^ 10 µL sample dispersion (200 µg mL^−1^) and 10 µL glucose solution (5 mg mL^−1^) were added to the wound surface. The wound of each group was treated with NIR irradiation for 10 min every day up to 7 days and the dark as control. After the treatment, wound tissue fluid was extracted and placed in 1 mL sterile normal saline. The number of bacteria was determined by plate method after incubating at 37 °C for 12 h. The wound area was recorded at days 1, 3, 5, and 7. The mice were sacrificed on day 7 and wound tissues were collected for histological analysis including H&E staining, masson staining, TNF‐*α* staining, CD34 staining, and VEGF staining.

### Statistical Analysis

SPSS software (IBM Corp., Armonk, NY, USA) was used for statistical analysis. One‐way analysis of variance (ANOVA) was used following the Tukey's post hoc test. Data were presented by mean ± SD.

## Conflict of Interest

The authors declare no conflict of interest.

## Author Contributions

M.H. contributed to methodology, data curation, and writing – original draft. Z.W. contributed to investigation and formal analysis. H.Y. and Q.W. contributed to validation. D.X. and X.P. contributed to visualization. Y.K.C., W.Y., G.Y., Y.D., and D.S. contributed to conceptualization, writing – review and editing, funding acquisition, and project administration.

## Supporting information

Supporting Information

## Data Availability

The data that support the findings of this study are available from the corresponding author upon reasonable request.
