# Supplementary material for: Multi‐Functional Bio‐HJzyme: Revolutionizing Diabetic Skin Regeneration with its Glucose‐Unlocked Sterilization and Programmed Anti‐Inflammatory Effects
Source: Adv Sci (Weinh). 2023 May 10;10(21):2300986. doi: 10.1002/advs.202300986 (PMC10375093; doi:10.1002/advs.202300986)
Supplement: Supplementary file 1 — Supporting Information [file ADVS-10-2300986-s001.pdf]

ADVANCED SCIENCE
Open Access

Supporting Information

for *Adv. Sci.*, DOI 10.1002/advs.202300986

Multi-Functional Bio-HJzyme: Revolutionizing Diabetic Skin Regeneration with its Glucose-Unlocked Sterilization and Programmed Anti-Inflammatory Effects

Miaomiao He, Zuyao Wang, Hang Yang, Qiancun Wang, Danni Xiang, Xinyan Pang, Yau Kei Chan, Dan Sun, Guangfu Yin, Weizhong Yang and Yi Deng**

Supporting Information

Multi-Functional Bio-HJzyme: Revolutionizing Diabetic Skin Regeneration with Its Glucose-Unlocked Sterilization and Programmed Anti-Inflammatory Effects

Miaomiao He, Zuyao Wang, Hang Yang, Qiancun Wang, Danni Xiang, Xinyan Pang, Yau Kei Chan, Weizhong Yang, Guangfu Yin, Yi Deng, Dan Sun

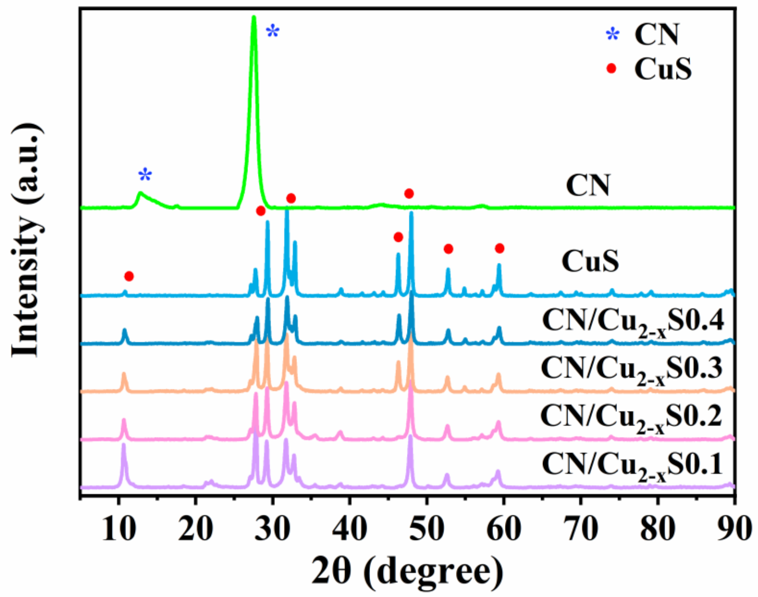

Figure S1. XRD spectra of samples.

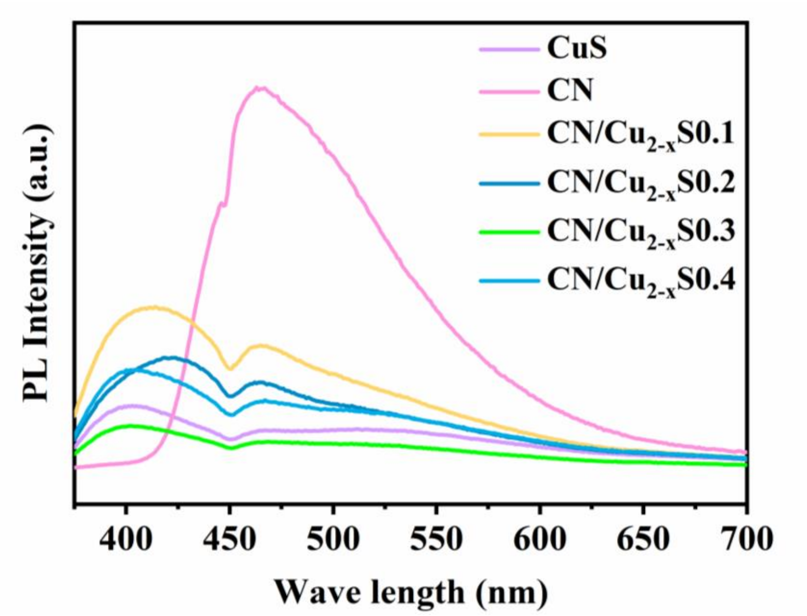

Figure S2. PL spectra of samples.

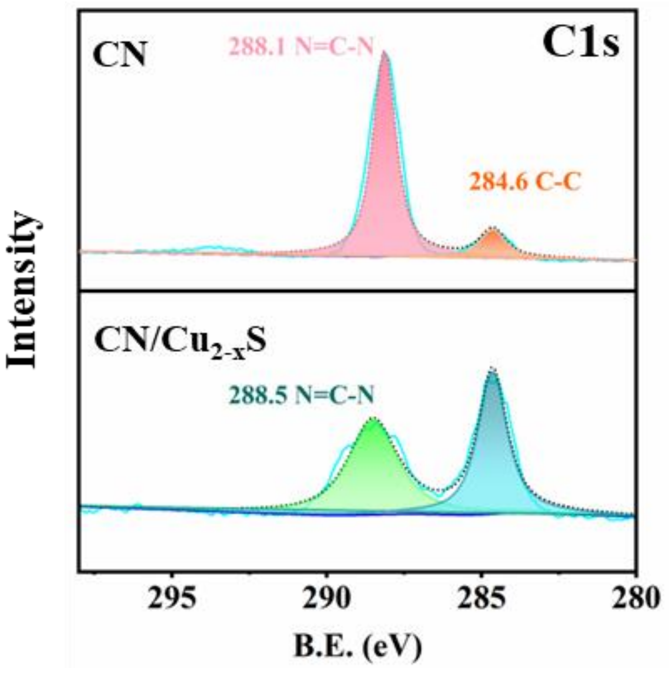

Figure S3. The deconvolution of C1s in XPS analysis.

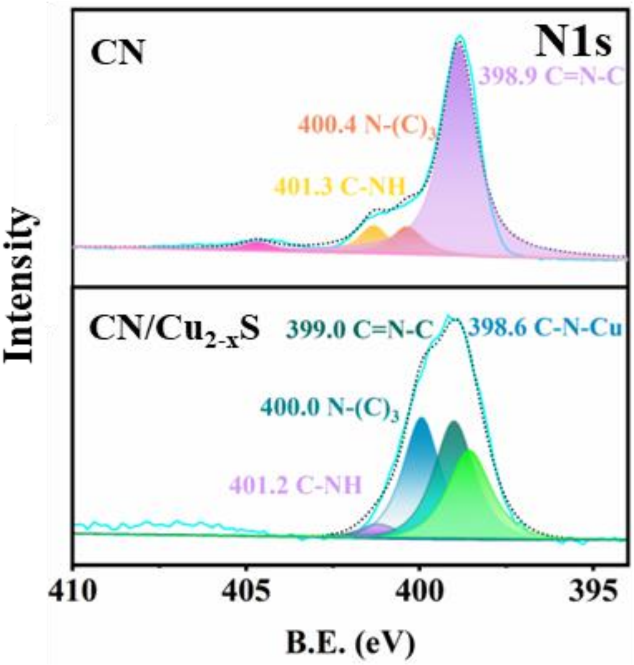

Figure S4. The deconvolution of N1s in XPS analysis.

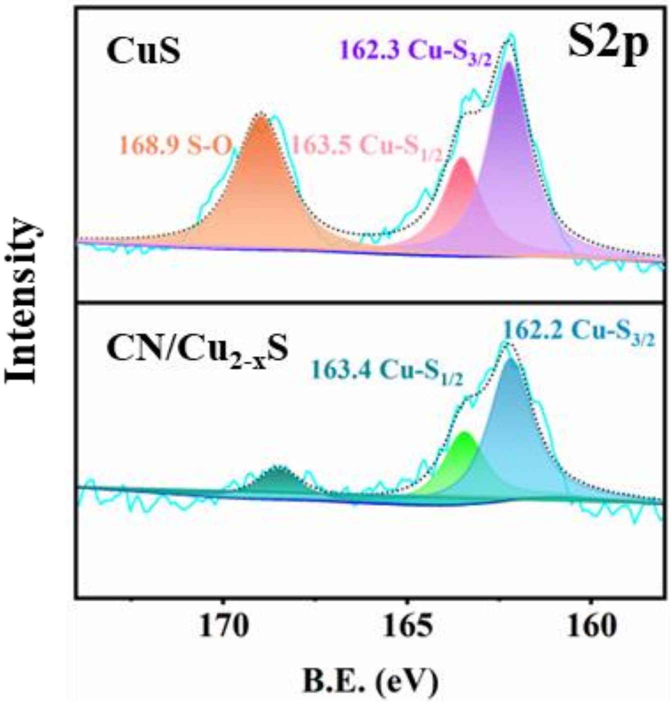

Figure S5. The deconvolution of S2p in XPS analysis.

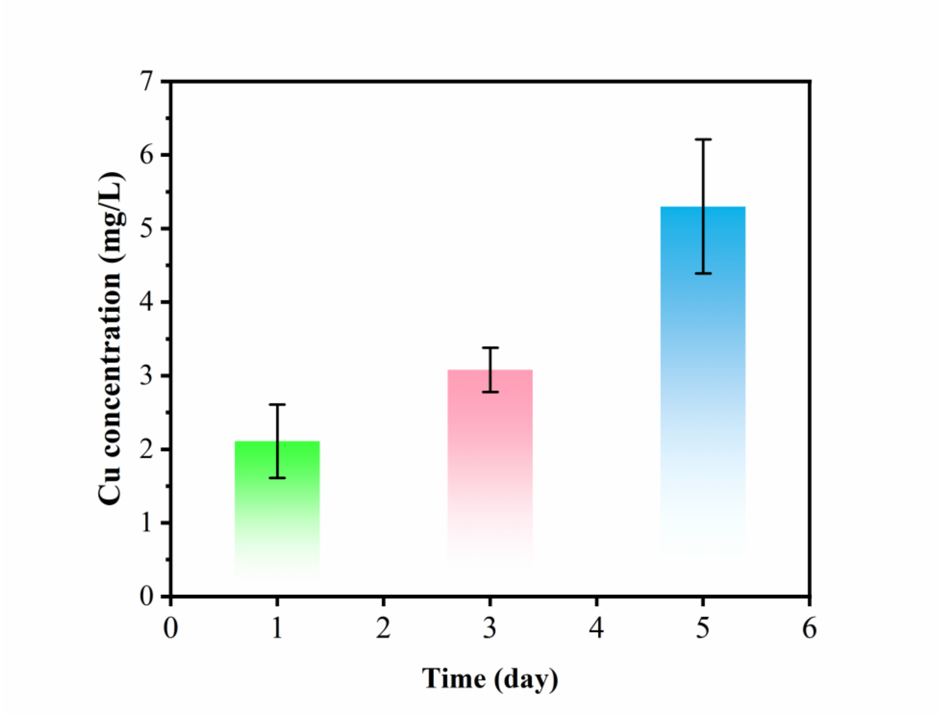

Figure S6. Cu concentration tested by ICP-OES on day 1, 3 and 5 (One-way ANOVA and Tukey's post hoc test, n=3).

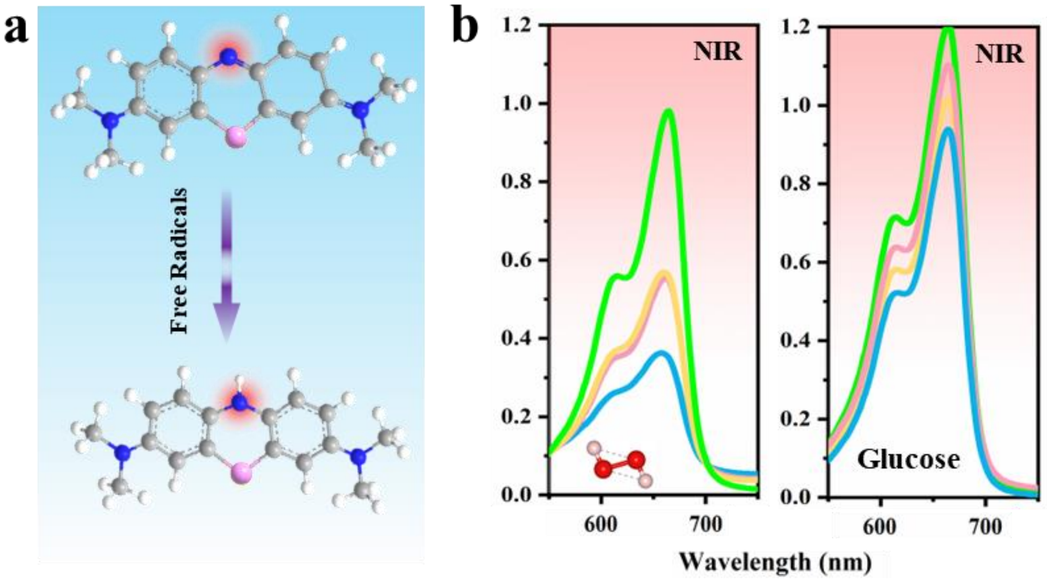

Figure S7. (**a**) Illustrative diagram of chemical reaction of MB oxidation; (**b**) UV-vis absorption spectra of MB reduction with NIR irradiation in H_2O_2 and glucose respectively.

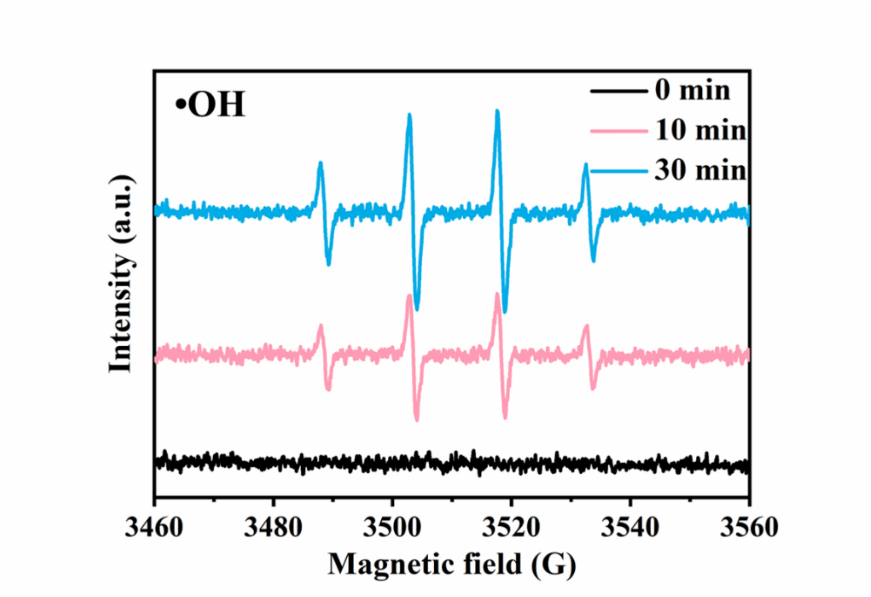

Figure S8. The ESR spectra of •OH generation of CN/$Cu_{2-x}S$.

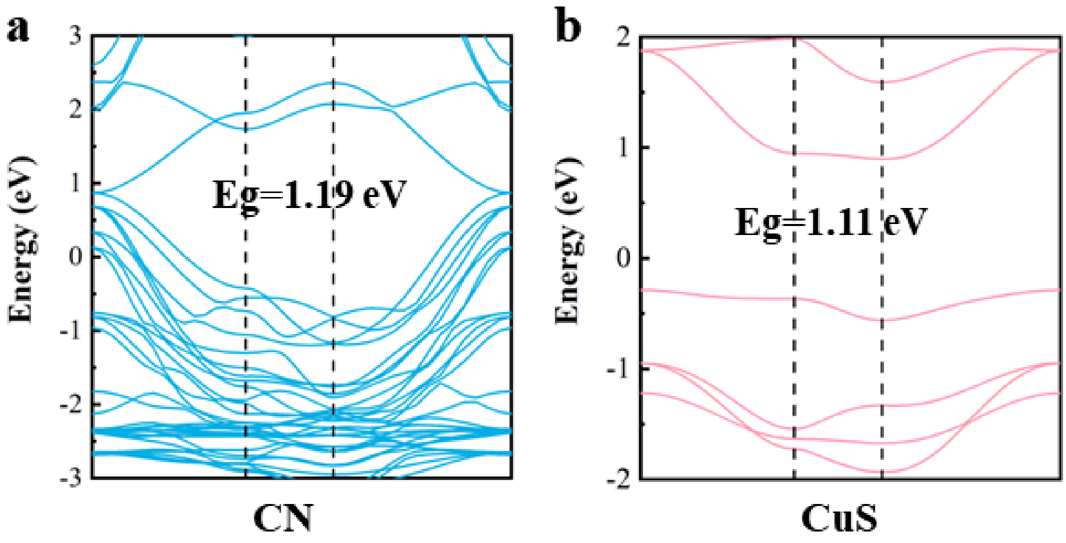

Figure S9. Calculated electronic band structures of unit cell of CN and $Cu_{2-x}S$.

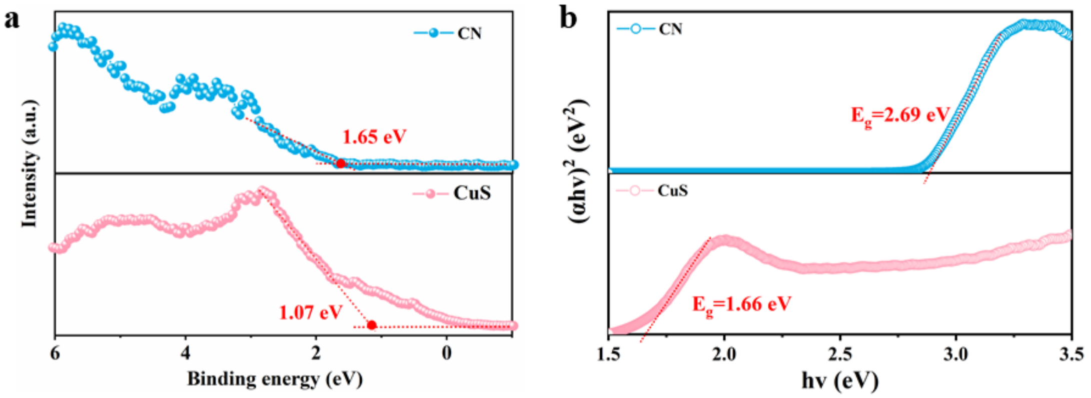

Figure S10. (**a**) The valence band (VB) analyzed by XPS of CN and CuS; (**b**) The band gaps of CN and CuS calculated from UV-Vis-NIR spectra.

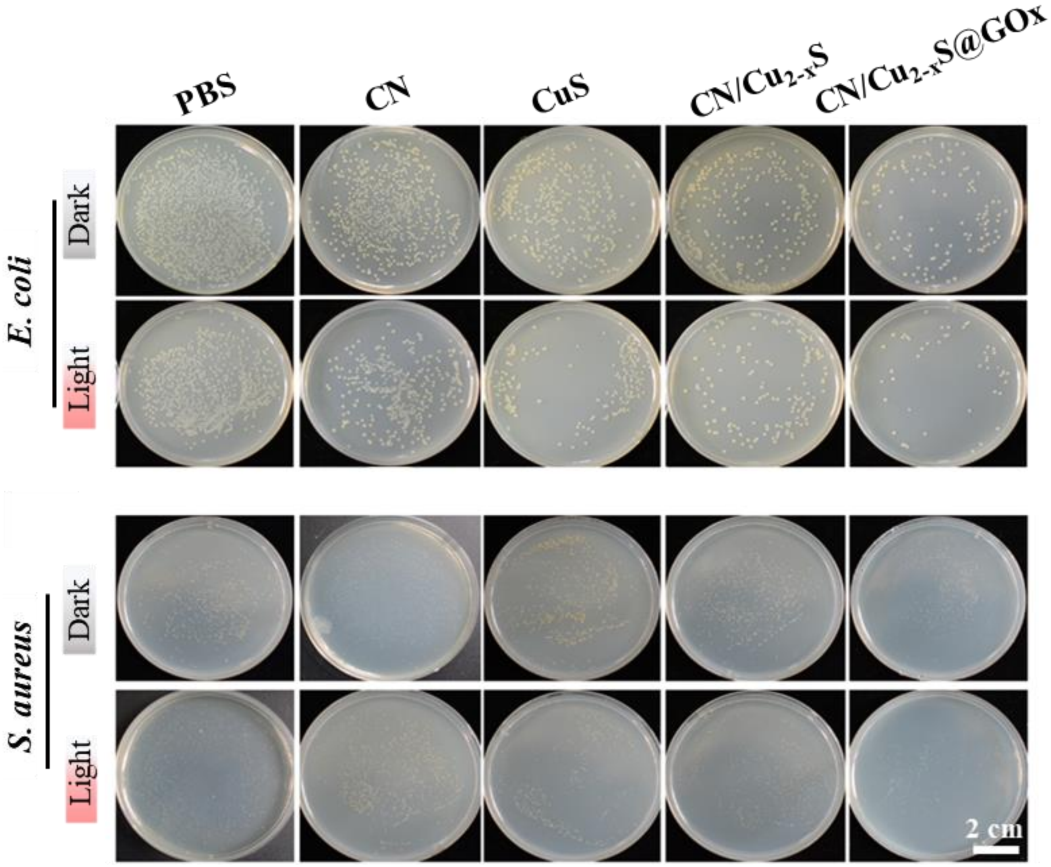

Figure S11. Images of (**a**) *E. coli* and (**b**) *S. aureus* colonies on diverse samples with/without NIR irradiation respectively.

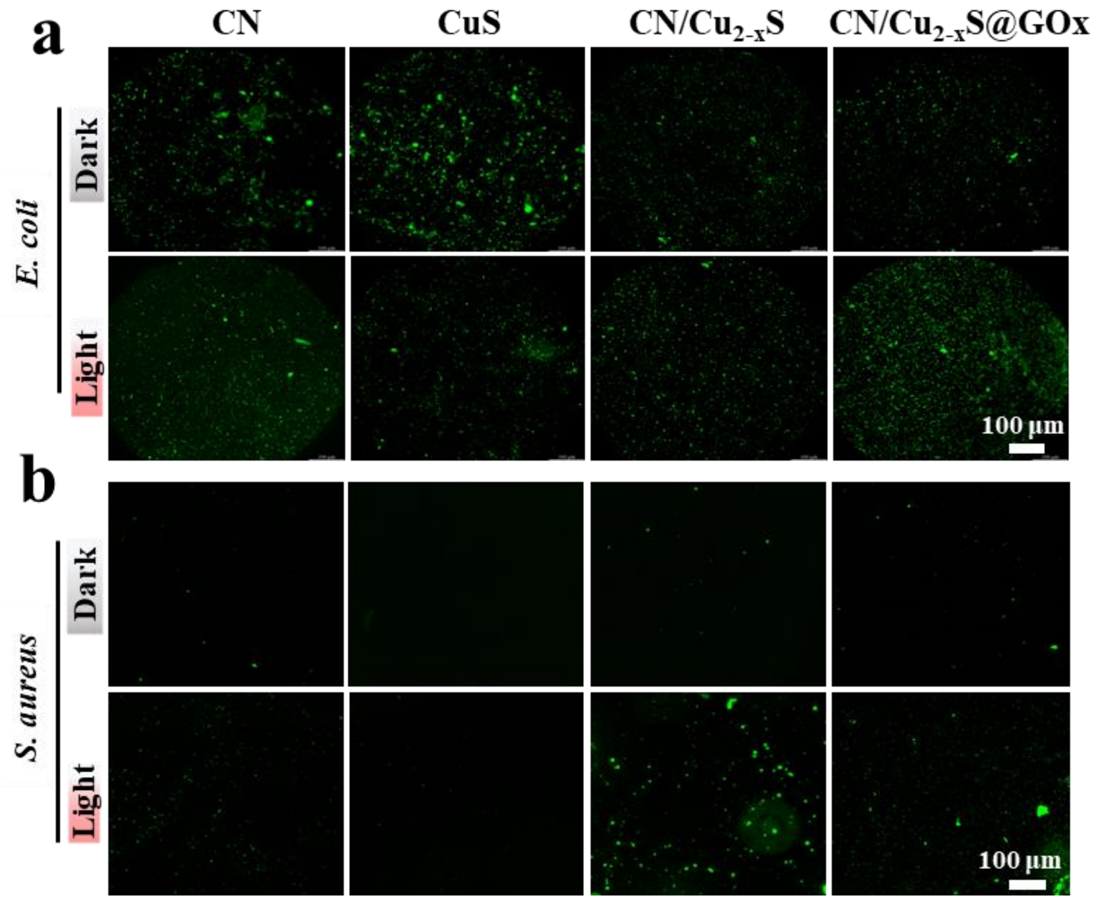

Figure S12. The images of intracellular ROS intensity in bacteria detected by DCFH-DA.

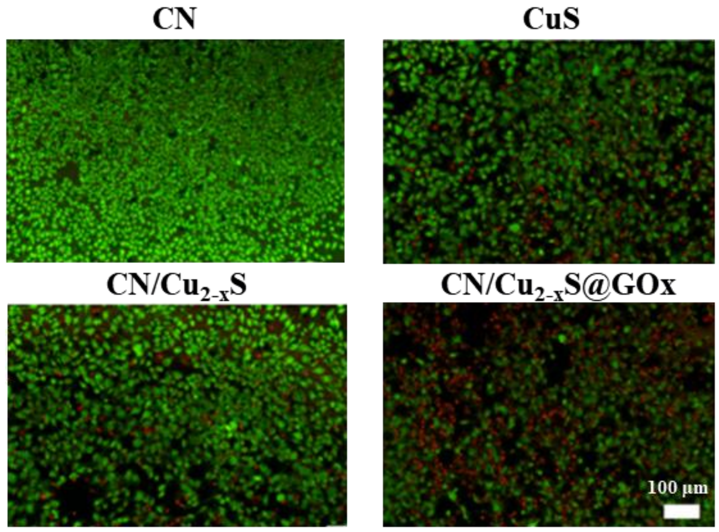

Figure S13. Live/Dead staining of L929.

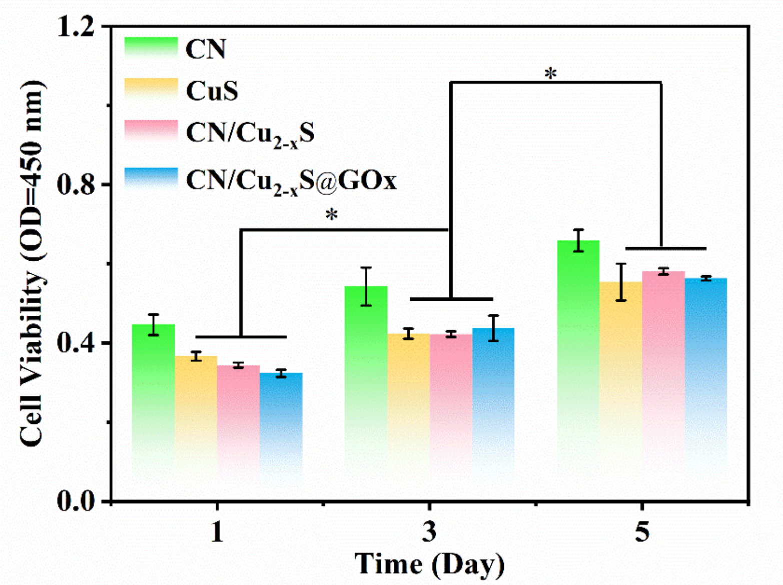

Figure S14. The quantitative CCK-8 results showed the cell viability (One-way ANOVA and Tukey's post hoc test, n=3, $*p < 0.05$).

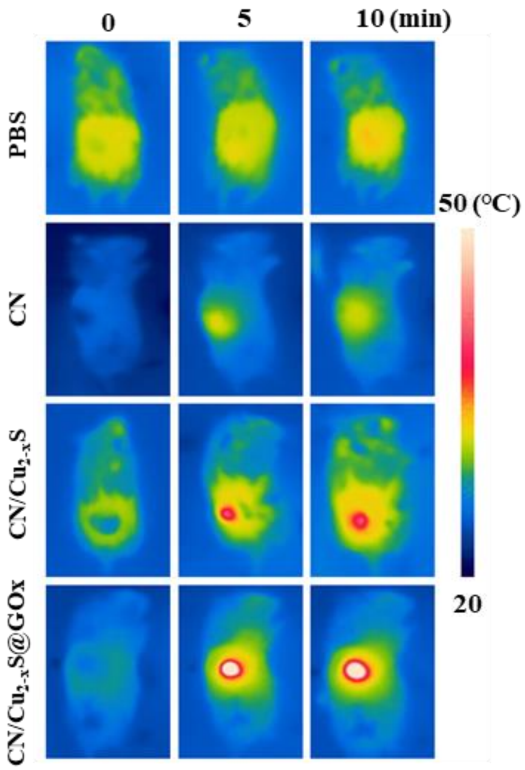

Figure S15. The photothermal images of each group for 10 min NIR irradiation.

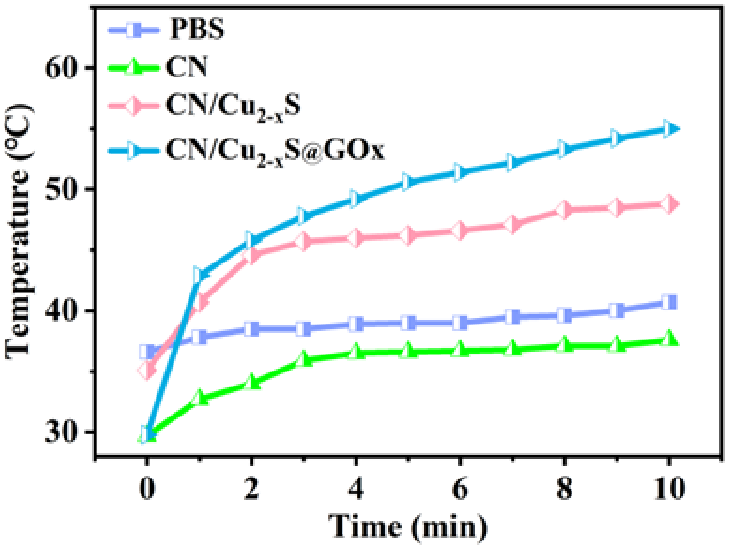

Figure S16. The photothermal curves of each group for 10 min NIR irradiation.

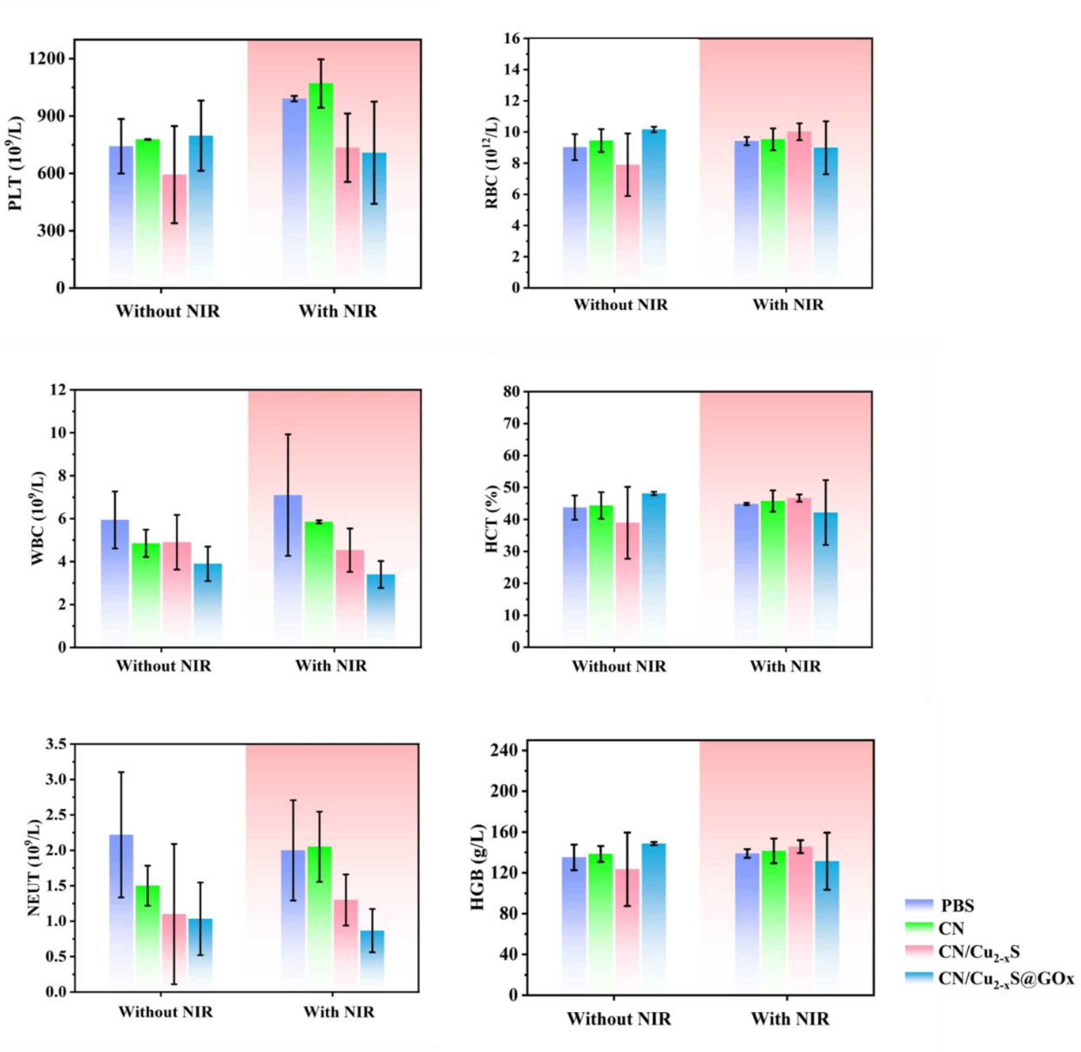

Figure S17. Blood cell analyzer test.

Table S1. The comparation of various POD-mimetic systems.

Nanozymes	Methods	Turnover number (S^{-1})	Vmax (M/s)	References
Fe_3O_4 ~ 40 nm	Hydrothermal method and *in-situ* deposition	3.18×10^{-5}	0.123×10^{-3}	[1]
FeN_4	Electrochemical exfoliation and *in-situ* deposition	0.63×10^{-5}	1.57×10^{-6}	[2]
FeN_5 SAzyme	A melamine-mediated two-step pyrolysis strategy	3.92×10^{-5}	8.47×10^{-6}	[3]
CH-Cu	Hydrothermal method	3.2×10^{-3}	1.22×10^{-10}	[4]
Fe-Art M	Hydrothermal transformation method and *in-situ* coating and carbonization of metal-phenanthroline complex	74.9×10^{-3}	11.51×10^{-8}	[5]
V-Art M		27.3×10^{-3}	5.14×10^{-8}	
Fe-N-C SAzyme	*In-situ* deposition	1.10×10^{-2}	1.18×10^{-7}	[6]
Pt hollow nanodendrities	Chemical deposition and etching	14.14×10^{-3}	/	[7]
CuO	Sol-gel method followed by annealing	2.23×10^{-3}	/	[8]
Cu NPs/N-C	KCl-template strategy	3.30×10^{-3}	/	[9]

References:

[1] F. Yu, Y. Huang, A. J. Cole, V. C. Yang, *Biomaterials* **2009**, 30, 4716.

[2] H. L. Fei, J. C. Dong, Y. X. Feng, C. S. Allen, C. Z. Wan, B. Volosskiy, M. F. Li, Z. P. Zhao, Y. L. Wang, H. T. Sun, P. F. An, W. X. Chen, Z. Y. Guo, C. Lee, D. L. Chen, I. Shakir, M. J. Liu, T. D. Hu, Y. D. Li, A. I. Kirkland, X. F. Duan, Y. Huang, *Nat Catal* **2018**, 1, 63.

[3] B. Xu, S. Li, L. Zheng, Y. Liu, A. Han, J. Zhang, Z. Huang, H. Xie, K. Fan, L. Gao, H. Liu, *Adv Mater* **2022**, 34, 2107088.

[4] J. Wang, R. Huang, W. Qi, R. Su, B. P. Binks, Z. He, *Appl Catal B-Environ* **2019**, 254, 452.

[5] Y. Long, L. Li, T. Xu, X. Wu, Y. Gao, J. Huang, C. He, T. Ma, L. Ma, C. Cheng, C. Zhao, *Nat Commun* **2021**, 12, 6143.

[6] Y. Feng, J. Qin, Y. Zhou, Q. Yue, J. Wei, *J Colloid Interf Sci* **2022**, 606, 826.

[7] C. Ge, R. Wu, Y. Chong, G. Fang, X. Jiang, Y. Pan, C. Chen, J. J. Yin, *Adv Funct Mater* **2018**, 28, 1801484.

[8] X. Wang, X. J. Gao, L. Qin, C. Wang, L. Song, Y. N. Zhou, G. Zhu, W. Cao, S. Lin, L. Zhou, K. Wang, H. Zhang, Z. Jin, P. Wang, X. Gao, H. Wei, *Nat Commun* **2019**, 10, 704.

[9] Y. Wu, J. Wu, L. Jiao, W. Xu, H. Wang, X. Wei, W. Gu, G. Ren, N. Zhang, Q. Zhang, L. Huang, L. Gu, C. Zhu, *Anal Chem* **2020**, 92, 3373.
